# Keeping Connected With School: Implementing Telepresence Robots to Improve the Wellbeing of Adolescent Cancer Patients

**DOI:** 10.3389/fpsyg.2021.749957

**Published:** 2021-11-12

**Authors:** Thomasin Powell, Jennifer Cohen, Pandora Patterson

**Affiliations:** ^1^Canteen Australia, Sydney, NSW, Australia; ^2^School of Women’s and Children’s Health, University of New South Wales, Sydney, NSW, Australia; ^3^Faculty of Medicine and Health, The University of Sydney, Sydney, NSW, Australia

**Keywords:** telepresence, robots, adolescent, cancer, education

## Abstract

**Background:** Adolescent cancer patients experience considerable absence from their education, contributing to poorer academic attainment and isolation from peers, and impacting wellbeing. Telepresence robots have been used to support the educational and social needs of young people with chronic illness. This article presents the results of the development and pilot-testing of a telepresence robot service in schools for adolescent cancer patients – the TRECA (Telepresence Robots to Engage CAncer patients in education) service.

**Methods:** Phase I used semi-structured interviews (*n* = 25) to assess the views of patients, parents, schools and clinicians on the benefits, acceptability, barriers, and enablers of utilizing robots in schools for adolescent cancer patients. Results from Phase I informed the development of the TRECA service. Phase II used semi-structured interviews (*n* = 22) to assess the implementation experiences of adolescent cancer patients, and their families, schools, and keyworkers who pilot-tested the TRECA service.

**Results:** Phase I demonstrated the need for telepresence technology in connecting adolescent cancer patients to school. Given the variable support during treatment, a telepresence robot service was considered an acceptable method of facilitating a school-patient connection. The recommendations provided in Phase I, such as the need for provision of ongoing education, training, and support to the patient and school, informed the development of the TRECA service. In Phase II, the themes of *The necessity of stakeholder buy-in*, *A facilitator of meaningful connection*, and *One size does not fit all* were generated. The TRECA service’s flexibility in meeting the needs of its users helped facilitate meaningful connections. Participants reported that these connections provided patients an enhanced sense of agency and wellbeing. The importance of stakeholder buy-in and taking an individualized approach to service delivery were also highlighted. Stakeholder miscommunication and lack of knowledge were key aspects of implementation needing improvement as the service is rolled out on a larger scale.

**Conclusion:** Using telepresence robots to connect adolescents to school during cancer treatment was regarded as highly acceptable, facilitating peer and academic connection. By making stakeholder-recommended improvements to the TRECA service’s existing processes, the service will continue to grow in effectiveness and capacity.

## Introduction

### The Impacts of Illness and Cancer on Adolescents’ Education

Illness can have a significant impact during adolescence as the young person and their family face the acute and long-term stressors of diagnosis, treatment, and condition management (Compas et al., 2012). In adolescents facing illness, an area that can be markedly affected is the ability to attend and engage with school. Compared to healthy peers or population norms, chronically ill students experience higher rates of absenteeism (Lum et al., 2017, 2019). They are also almost four times as likely to have academic challenges than healthy peers (Lum et al., 2019). Furthermore, greater illness and treatment side-effect severity have been associated with poorer academic performance, grade repetition and reduced educational attainment (Lum et al., 2017). Beyond academic outcomes, illness can also significantly impact students’ psychosocial wellbeing. For instance, chronically ill students are more than twice as likely to be experiencing moderate to high levels of emotional distress, and almost five times more likely to have low social confidence than their peers (Lum et al., 2019).

Young people with cancer experience even more absences from school than those with any other chronic condition (Vance and Eiser, 2002). A diagnosis of cancer during adolescence can significantly interfere with an adolescent’s quality of life in all spheres, including physical health, social support, wellbeing and self-perception, and the family environment (Kim et al., 2016; Penn et al., 2016). Although cancer experiences can vary widely, young people often experience long and difficult treatment periods with side effects such as reduced physical energy, changes in physical appearance, and pain and discomfort (Kim et al., 2016; Penn et al., 2016). This can result in long periods of recovery spent in hospital or at home (Penn et al., 2016). Consequently, many young people have prolonged or frequent periods of absence from school (Tsimicalis et al., 2018), which can lead to poorer academic attainment (Bonneau et al., 2011) and impact friendships (Barrera et al., 2005; Winterling et al., 2015). Survivors of childhood cancer are also more likely to need to repeat a school year compared to siblings (Bonneau et al., 2011) and matched population controls (Barrera et al., 2005), with diagnosis occurring during secondary school being a risk factor (Barrera et al., 2005; Bonneau et al., 2011). Feelings of loneliness and isolation from their school community and peers are also common for young cancer patients (Searle et al., 2003; Boles et al., 2017), along with experiencing anxiety about reintegrating with their peer network at the end of their treatment (Pinquart, 2017; Collins et al., 2019). Bullying is additionally a risk factor when they do return to school (Choquette et al., 2016).

### Educational Support for Adolescents With Cancer

Given the social isolation experienced by chronically ill students, it has been suggested that schools complement academic support with additional practices focused on supporting patients’ general engagement with school and promoting a sense of belonging (Leigh et al., 2016; Lum et al., 2017). Indeed, research suggests that maintaining regular contact with peers during periods of absenteeism may facilitate a smoother transition back into the classroom and schoolyard (Choquette et al., 2016). This regular contact enables young people to feel more positive about being in school and reduces apprehension about their return (Wilkie, 2012; Zhu and Van Winkel, 2014). As such, programs and services that connect young patients to the school environment whilst they are unable to attend in-person may improve wellbeing whilst they are away and protect against some of the negative outcomes of absenteeism. Given the physical, social, psychological, and academic impacts, it has been suggested that an integrated and multidisciplinary approach involving teachers, healthcare professionals, psychologists, and the family should be taken when schools support a student with a chronic illness such as cancer (Shaw et al., 2010). Furthermore, research has highlighted the value of individualized education plans and school re-entry programs that tailor school support to the specific needs of the patient and extend beyond educational outcomes by also supporting patients’ psychosocial and physical needs (Leigh et al., 2016; Lum et al., 2017).

### Information and Communication Technologies

Information and communication technology (ICT) is increasingly used to support young people to attend their classrooms and supplement the exchange of work between the school and patient (Hopkins et al., 2014). A diverse range of ICT methods have been trialed to support children and adolescents who experience long term absence from school due to chronic illness. These ICT methods have included BlackBerry devices (Fels et al., 2003), a telepresence app (Hopkins et al., 2014), videoconferencing facilities (Ellis et al., 2013), and ambient technologies (Wadley et al., 2014). The few case studies which have been conducted suggest that ICT can assist children and adolescents with chronic conditions to keep in touch with friends at school and support wellbeing (Nisselle et al., 2012; Ellis et al., 2013; Wadley et al., 2014). Although these preliminary findings appear promising, some classroom teachers found the technology caused distraction in the classroom (Ellis et al., 2013).

Telepresence robots have been more recently used to support the education of children with chronic conditions (Page et al., 2020). Telepresence robots are remote-controlled devices with wireless connectivity allowing both video and audio connection, and can be mobile or stationary (Page et al., 2020). There have been very few studies assessing the acceptability of telepresence robots in schools for adolescent cancer patients. Looking at their effects on chronically ill students generally, a recent scoping review and thematic analysis examined the potential utility of telepresence robots for Australian schools, suggesting that telepresence robots can facilitate positive educational experiences and social development, reducing isolation (Page et al., 2020). This review also highlighted the issues of connectivity difficulties and privacy potentially reducing robot acceptability, and reported instances of lack of acceptance of the technology by school peers, leading to bullying (Page et al., 2020). A recent trial of telepresence robot technology with chronically ill students found that participants reported perceived increased connectedness and improved mood following the use of a telepresence robot (Chubb et al., 2021). Finally, a recent study by Weibel et al. (2020) specifically examined the impact of desktop telepresence robots with a one-way camera and two-way audio communication capabilities on children and adolescents with cancer. The authors found that the robot facilitated social and academic connection, allowing patients to feel included in their learning community, and reducing loneliness and patients’ perception of being academically behind (Weibel et al., 2020).

### The Current Study

**FIGURE 1:**
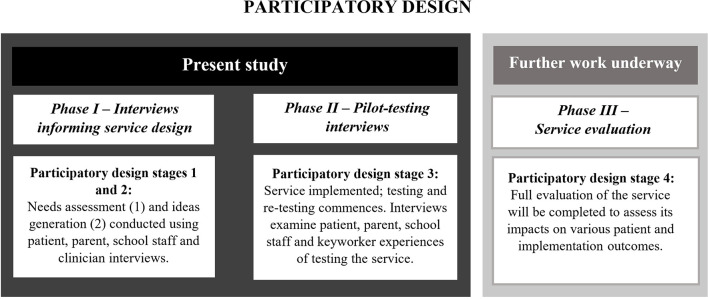
Participatory design (PD) elements encompassed by each study phase.

In 2017, Canteen^1^ began exploring the development of a telepresence robot service. This paper presents the results of a two-phase study on the development and pilot-testing of telepresence robots in schools for adolescent cancer patients. To maximize the chances of successfully developing and implementing a telepresence robot service, a participatory design (PD) approach was chosen (Clemensen et al., 2007) with users involved in each phase of the development of the service (Figure 1). PD allows users to be involved in the design and testing of a technical healthcare solution (Clemensen et al., 2017). This study represents the first three stages of the PD approach (Clemensen et al., 2017) outlined in “Phase I” and “Phase II” of Figure 1 below, with stage four (outlined in “Phase III” of Figure 1) currently underway.

Phase I focused on *needs assessment* and *ideas generation* and aimed to assess the views of patients, parents, schools and healthcare professionals of the benefits, acceptability, barriers, and enablers of utilizing robots in schools for adolescent cancer patients. Results from the Phase I study were used to inform the design and implementation of the TRECA (Telepresence Robots to Engage CAncer patients in education) service. Phase II focused on the *pilot-testing* stage of PD and aimed to assess the implementation experiences of young cancer patients, and their families, schools, and keyworkers. Similar methodologies have been successfully utilized by other research groups to design and implement ambient technologies for chronically ill children (Wadley et al., 2014).

## Phase I: Needs, Acceptability, Barriers and Enablers for a Telepresence Robot Service

### Method

#### Design

Phase I was a qualitative study using semi-structured interviews to assess the views of patients, parents, schools and clinicians. It comprised the first two stages of PD: (1) needs and acceptability of telepresence robots (needs assessment), and; (2) barriers, and enablers (ideas generation) of utilizing telepresence robots in schools for adolescent cancer patients. The study protocol for Phase I was approved by The University of Sydney Ethics Committee [2017/770]. Telepresence robots were not utilized in Phase I.

#### Participants

To optimize the success of our future robot service, we conducted stakeholder interviews with the following key groups who were deemed essential for the implementation of a robot service: (1) Young people who were either currently receiving or had completed active treatment for cancer whilst they were in secondary school; (2) Parents/guardians of individuals who were receiving or had completed active treatment for cancer whilst they were in secondary school; (3) Individuals working in a teaching profession; and (4) Healthcare professionals working with adolescents with cancer. In total, 25 participants were recruited to Phase I: healthcare professionals (*n* = 8), schoolteachers (*n* = 8), adolescent cancer patients (*n* = 7), and parents of adolescent cancer patients (*n* = 2). None of the respondents were linked except for one patient/parent dyad. Most participants were female (*n* = 20), and participants were from several states across Australia including New South Wales (*n* = 8), Victoria (*n* = 10), South Australia (*n* = 4), Queensland (*n* = 2) and Tasmania (*n* = 1).

#### Procedure

Adolescent patients and parents of adolescent patients were recruited *via* invitations sent by Canteen psychosocial clinicians and through social media advertising. Teaching staff from a diverse range of school settings (e.g., public, private, and Catholic) and with a range of experiences in various locations of Australia were recruited through social media advertising. Healthcare professionals were purposively sampled from AYA cancer services and pediatric hospitals for their experience in working with adolescent cancer patients.

#### Interviews

The semi-structed interview guide was developed by a multi-disciplinary group of researchers and clinicians (Supplementary Appendix 1). The semi-structured interview focused on the needs assessment and ideas generation stages of PD. The needs assessment questions focused on experience of schooling during treatment, schooling and academic support during cancer treatment, peer support, use of technology for schooling during treatment and experience of returning to school. In addition, the parents and healthcare workers were asked their views on the perceived impact of being hospitalized on an adolescent’s wellbeing. In the ideas generation phase of the interviews, participants were asked about their views on the idea of a robot program for adolescents going through cancer treatment and perceived barriers and enablers to using a telepresence robot during treatment. The semi-structured interviews were flexible in nature and built upon the ideas, experiences, and needs brought up by the participants. Interviews were completed over the telephone by an experienced researcher. Interviews were audio-recorded to allow accurate verbatim transcription of the interview.

#### Analysis

Data were analyzed using content analysis as guided by Miles and Huberman (1994). Content analysis methodology involved the development of a coding scheme which was then applied to topics of interest within the transcripts. The interviews were analyzed inductively line-by-line and the codes were placed into emerging topics. The coding framework was informed by the data set and refined by the researchers to further data analysis. The data was coded by two researchers experienced in qualitative research.

### Results

#### Stage 1: Needs Assessment

In the needs assessment phase of the interviews, two themes emerged: inconsistency of educational support during and after cancer treatment, and the impact of cancer on patient isolation and wellbeing (Table 1).

**TABLE 1:** Needs assessment and ideas generation themes.

**Identified theme**	**Description**
Inconsistency of educational support during and after cancer treatment	Cancer treatment impacted school attendance, with supports provided variable. The impacts on education continued once treatment had completed with many adolescent patients finding it difficult to reintegrate back into the school environment.
Impact of cancer on isolation and wellbeing	Missing impacted social connections, isolating patients from their peers for an extended period.
Telepresence robots can support adolescents’ education and isolation during cancer treatment	A telepresence robot was seen as a novel way to engage adolescents with their education and their peers during cancer treatment, facilitating reengagement back with school at the completion of treatment.
Assessing suitability of the robot for a young person	It is important to screen a young person prior to implementation of a robot to ensure they are suitable for the service.
Need for the school to be engaged in the process	It was important that education on the telepresence robot program is provided to the school and the young person’s peers prior to, and during implementation of a robot.
Ensuring good user experience with the appropriate technology	The school must have the technical capacity to host a robot and ongoing IT support is required throughout the program.

##### Inconsistency of Educational Support During and After Cancer Treatment

Throughout the interviews, participants explained the impacts that cancer treatment had on the education of young patients and how well the education system could manage this. Participants often reported young people missing out on their education due to complex treatment regimes, treatment side effects and neutropenia. For many young people, attending school during treatment was a priority for them at the beginning of treatment. Despite the willingness of young people to attend school they could not always attend due to treatment side effects.

*“In the beginning I was very persistent that I wanted to go to school*… *but with the treatment came tiredness and energy loss. In the middle [of treatment] I was reluctant to go [to school]*… *because I didn’t have enough energy to get out of bed”* Patient

Throughout the cancer journey, there were often occasions when parents or teachers believed that the young person was well enough to attend school but did not encourage them to attend, as staying rested and focusing on their health was seen to be a bigger priority. It was common for students to fall behind at school considerably during the treatment period, but there also appeared to be a longer-term impact of treatment on their ability to stay in school. For young people who were able to attend school during treatment, families acknowledged that their attendance was more about peer connection than learning.

*“If we were lucky we could get him to school once a month maybe. It was basically more for social [reasons] than really doing any work. Just to keep him engaged”* Parent

For patients who spent a lot of time in hospital, some hospital services had specialized positions whose role it was to facilitate the connection between the school and the patient. For some young people, there were supports in the form of tutoring, available through non-government organizations or hospital school programs. In noting the tutoring based supports available to young cancer patients, one healthcare worker stated “…*But one concern that comes up for a lot of families is that disconnect from school*…*I think connection is so important*…”

The way in which patients engaged with the education system during their treatment was variable. Some schools put systems in place to support students to continue with their education during treatment by speaking on the phone with the young person or sending work home. Some schools also facilitated ongoing peer connection with the students from the school and some participants suggested that facilitating peer connection during treatment improved the likelihood that a young person would attend school during treatment.

*“*…*make sure [the patient] is engaged [with the school], making sure they stay in contact with their friends, making sure they understand what’s happening day to day at the school. It’s that everyday information that if you’re not there for 2 weeks and you walk back in, that’s an awful feeling”* Healthcare Professional

However, the education system was often reported to be inconsistent in the support provided to young people being treated for cancer. In some instances, young people relied on their friends providing the work and assignments from school.

*“I always asked my friends, if the teachers give us anything just send it to me so I can try and at least do it”* Patient

Although a few students returned to school in a full-time capacity following treatment, most had a slow reintegration process, receiving modified work or alternate work assignments. For patients who missed quite a bit of school due to treatment, there was concern around going back to school and reintegrating with the school community.

*“I sort of actually dreaded going back to school. In Year 9, [be]cause I sort of thought, it was virtually the first day of school all over again”* Patient

Another identified gap in the system was when treatment was completed and where educational supports from the hospital often stopped.

*“.If you asked families and young people where the gaps probably are, it’s possibly down the track when they’re off treatment and, you know, not at the hospital anymore. I imagine that’s probably where there are still gaps”* Healthcare worker

If patients had some connection with their school during treatment, they often found it easier to reintegrate back to school once treatment is completed.

“*There [are] definitely young people who are really motivated to stay connected and [for] some other young people their motivation to connect with school is not the same. So then the re-entry*… *can be really hard for them*” Teacher

##### Impact of Cancer on Isolation and Wellbeing

Adolescence was considered a challenging time to have a cancer diagnosis as peer development was key during this time. Participants acknowledged that being diagnosed with cancer as an adolescent was particularly isolating as it removed young people from their usual friendship groups. Young people noticed this more when they returned to school.

*“I felt like everyone had made their friendship groups and connections. In some ways it’s like a new student coming in.’* Patient

For many young people, the biggest impact from missing school during treatment was missing out on all the experiences they would normally have with their peers.

*“I missed out on [camp] because I was sick. The stories and stuff that happened on the program, people got a lot closer*… *I had to sit out of it, which sucked a fair bit”* Patient

Participants highlighted the considerable impact which missing school could have on a young person’s wellbeing. Increased anxiety was frequently mentioned, and some participants spoke of bullying and depression.

*“One of the big things in youth cancer is that they become isolated from their friends and their peers and it can lead to depression”* Healthcare Worker

*“I used to feel down that I wasn’t able to go to school. I guess it did upset me”* Patient

Participants suggested that maintaining social connections during cancer treatment may help improve the wellbeing of young patients.

*“One day [the young person] was laying down trying to sleep because that is all he wanted to do while he was [in hospital]. Then his friends rang him and in about 20 min he was sitting up in his hospital bed with a smile, chatting and laughing. That made a really big impact”* Healthcare Worker

#### Stage 2: Ideas Generation

During the interviews, participants were given an opportunity to discuss their views and ideas on the telepresence robot service and the barriers and enablers that would need to be addressed to ensure a successful service implementation. Participants considered telepresence robot technology to be appropriate for supporting an adolescent with their education and feelings of isolation during their cancer treatment. To facilitate a successful telepresence robot service, participants suggested: the need to assess whether the young person is suitable for the service, the need for the school to be engaged with the service throughout the implementation process, and the use of appropriate technology that ensures a good user experience.

##### Telepresence Robots can Support Adolescents’ Education and Isolation During Cancer Treatment

There was overwhelming support for the use of telepresence technology as a novel and fun way to concurrently engage adolescents in their education and with their peers. The robot was considered a more acceptable form of technology than the use of a tablet alone because the robot could move about and be controlled by the young person from hospital or home.

*“I think [the robots] are a really good idea as kids don’t feel alone, that they have to go through [treatment] alone. They have friends, they can connect with their school, keep in touch with their friends, get to know what’s going on at school’* Patient

Some patients reflected on the robot and how it may have facilitated an improvement in wellbeing if they had access to a robot during their treatment. The robot service was also seen as a way of providing patients’ friends with an understanding of the patient’s cancer experience, allowing their peers to develop social understanding, compassion, and empathy. The robot was also thought to facilitate reengagement with the school and peers when cancer treatment was completed.

*“*…*[the robot] gives their classmates an opportunity to have more of an understanding of what they are going through as well. So [the patient] is not having to come back to school after a few months off and be bombarded with questions and people not knowing how to speak to them as well.”* Healthcare worker

##### Assessing Suitability of the Robot for a Young Person

Participants suggested that some young people may not want to use the robot because they do not want to draw attention to themselves. This was emphasized by a statement from one of the patient participants:

*“I didn’t want the whole of my [school] cohort knowing that I was sick. I didn’t really want people to see me as sick.”* Patient

There were comments that not all patients would like the visibility of the robot, and the ability to have a choice of robot style or use of an avatar in place of the video stream would likely increase acceptance.

*“*…*maybe the first thing a [patient] could do is create an avatar*… *and that would allow them*…*to turn off the camera on their side, they would be able to show the avatar”* Patient

Participants suggested that screening of the young person occurs, prior to implementation of a robot, to ensure they are suitable for the service.

##### Need for the School to be Engaged in the Process

Participants suggested there would likely be some resistance and concern from teachers regarding the privacy associated with having this technology in the classroom for fear of being videotaped. This same concern about privacy was highlighted as a potential issue with parents as well.

*“a lot of teachers are really paranoid about being videoed*…. *some [teachers] are just concerned that that information might be videoed and then put on YouTube*…. *and make them look negative”* Teacher

Educators were concerned that having a robot in the classroom would be a distraction for other students, especially when the robot is first implemented. There was also concern about the nature of the physical school environment (i.e., stairs), making it difficult to maneuver a robot around the school to each classroom.

*“*…*the number of schools that I have worked across, I’d say 80% had stairs. Depending on what of these areas were connecting would kind of dictate the viability of the robot”* Teacher

Participants highlighted the importance of providing adequate education to the teachers and support to the school prior to implementation of a telepresence robot. Education on the robot service and information about cancer could be extended to include students in the patient’s year group and their parents.

*“It would be a matter of just talking to the school and*…*getting on board with [the school] to work out how it would work and how you are going to implement it and talking to the kids on how does it all work”* Parent

To support the young person to successfully use the robot within the school environment it was suggested there be a main contact person within the school community to be a point of contact and to take responsibility for the robot. This could be done in combination with a student, using a buddy system, as a way of teaching responsibility.

“*If there was a buddy system and the other student was to take on a role where they were responsible for [the robot] I think that’s a really good thing*” Teacher

##### Ensuring Good User Experience With the Appropriate Technology

The importance of having technology with good user-experience was emphasized consistently throughout the interviews, especially when using it in a busy classroom where teachers would have limited time to manage any technological issues which occurred. Many of the participants mentioned the lack of Wi-Fi available on school property and within the hospital.

*“*…*Because of our remoteness, the internet is a real issue for us*…*so that would be probably a very strong barrier”* Teacher

Schools would require screening at the start of the implementation robots to ensure adequate infrastructure is available within the school to support the telepresence robot service. The schools would also require ongoing support with technological issues to facilitate a successful implementation of the telepresence robots.

*“It will come down to how easy [the robot] is to set up and to start because sometimes technology can get a little bit too complicated and when that happens it will get pushed to the side.”* Parent

## Phase II: Pilot-Testing of the Telepresence Robot Service

### Service Features

**FIGURE 2:**
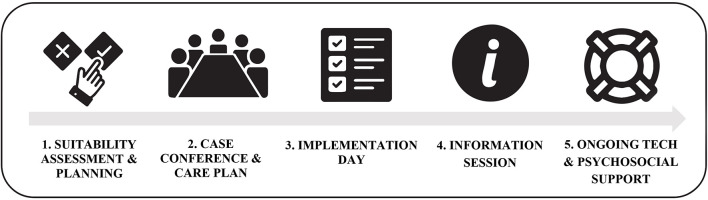
Components of and progression through the telepresence robot service.

The TRECA service (see Figure 2) was developed based on the findings from the needs assessment and ideas generation stages of the PD process. As part of the service, patients and schools are provided with both the robot itself and ongoing multi-domain support. To facilitate the assessment of the young person’s suitability for the robot’s service, upon referral to the service, patients are assigned a psychosocial support worker (keyworker) who can check in with them regularly and provide support as needed. Prior to implementation of the robot, the keyworker also works with the patient, their family, and the school to develop an implementation plan, defining when and how their robot will be used (e.g., which classes they wish to connect to, whether it is used at recess and lunch time). Upon a patient’s referral to the service, the patient’s school (usually the school principal, vice principal, or head of student wellbeing) is contacted by the TRECA service coordinator, who provides them with an overview of how the service runs. If the school indicates their potential interest in supporting their student to access the TRECA service, the school is then provided with a written information pack outlining the service and technology. To maintain ongoing engagement with the school, the school is provided with staff training on how to use and store the robot and continual IT support throughout the period in which they are hosting a robot. Additionally, upon the robot’s implementation in a school and with the consent of the patient, an information session (called When Cancer Comes Along; Wright et al., 2021) on the robot, cancer and its impacts, and where to get support, is offered to students in the same cohort as the patient. It is recommended to the school that the patient be assigned one or more “robot buddies” – close friends of the young person who can assist with the logistics of the robot each day (e.g., transport to and from class). The service coordinator oversees all components of the TRECA service to ensure communication is maintained between all parties involved, and that both the adolescent and school have a good user experience.

#### The Telepresence Robots

The TRECA service offers two types of robot to patients – the Double^TM^, and the Kubi^TM^, both of which enable patients to videoconference into their classroom/school. The Kubi^TM^ comprises an iPad held in a base that sits on a desk in the classroom. The base enables the user to swivel the iPad so that they can look around the room. The Double^TM^ robot comprises an iPad that is attached to a Segway base, enabling the user to remotely drive the robot around the school or classroom. Patients use a laptop or tablet (either their own, or one provided to them if needed) to connect to and control their robot from home or hospital. The robot connects either to a school’s Wi-Fi network, or a cellular network, depending on which is most practicable. Prior to robot implementation, the robots’ software’s recording/photo taking function is disabled for privacy reasons. The TRECA service is provided free of charge.

### Method

#### Design

In Phase II, a pilot-testing evaluation was conducted using semi-structured interviews to explore key stakeholders’ implementation experiences of using telepresence robots to enable adolescent cancer patients to attend school remotely during their cancer treatment. The pilot-testing comprised the third stage of PD: testing and retesting (Clemensen et al., 2017). The study was approved by the University of Sydney Ethics Committee [2018/160] and the University of New South Wales Ethics Committee [HC200043].

#### Participants

A total of 22 participants took part in Phase II of the study, including patients (*n* = 6), parents of patients (*n* = 6), school staff members (*n* = 5), and Canteen keyworkers (*n* = 5). Participants fell into one of four groups: (1) Adolescents with a diagnosis of cancer or a hematological condition, who were receiving cancer treatment or had completed treatment but were unable to continue with their education full-time either due to their diagnosis or the side-effects of treatment; (2) Parents/guardians whose child was part of the TRECA service; (3) School staff who worked closely with a young person and their family on the TRECA service; and (4) Psychosocial keyworkers who provided support to the young person and family as part of the TRECA service. All participants were linked to at least one other participant except for one keyworker.

#### Procedure

When a young person had used the TRECA service for a minimum of 3 months, they and their parent/caregiver were contacted *via* email or text message to invite them to participate in the study. Participants were followed-up by phone, email, or text message no more than two times. Participants were informed that consent was voluntary and that they could withdraw from the study at any time without penalty. Additionally, a parent or guardian was asked to provide consent through the return of a signed consent form for young people aged 12–17 years. If the young person agreed to participate, their parent/caregiver, keyworker, and school representative that were involved with the implementation of the robot were contacted. All participants received an information sheet and consent form (including participants aged 12–17, who signed to indicate assent).

#### Interviews

The semi-structured interviews were designed by a multi-disciplinary group of researchers and clinicians (Supplementary Appendix 2). The semi-structured interview explored themes such as connection to school, impact of cancer on school experience, maintenance of relationships, and continued education, and perceptions of the robot itself and TRECA service. The semi-structured interviews were flexible in nature and built upon the ideas, themes and experiences brought up by the participants. Interviews were completed over the telephone by an experienced researcher and lasted between 19.15 and 47.63 min (*M* = 30.71). Interviews were audio-recorded to allow accurate verbatim transcription of the interview.

#### Analysis

The data was analyzed using a reflexive approach to thematic analysis, as described by Braun and Clarke (2006); Braun et al. (2018). For Phase II, the six steps to thematic analysis outlined by Braun and Clarke (2006) were followed to guide analysis: (1) Familiarization with the data took place by reading transcripts multiple times and noting down initial ideas regarding emerging patterns; (2) Initial codes were generated for interesting or significant data points by systematically examining each transcript; (3) Codes were collated into meaningful themes; (4) Themes were reviewed for clarity and coherency at both a code and broader data set level; (5) Themes were named and further refined in accordance to the overall picture of the analysis; and (6) Themes were reported in the context of the research questions and evidence base, and unpacked using extracts as examples. Data were analyzed using NVivo; a qualitative tool which supports comprehensive coding.

### Results

Regarding participants’ experiences of using a robot to enable patients to attend school remotely, a total of three themes were generated from the qualitative data (Table 2).

**TABLE 2:** Phase II themes and subthemes.

**Theme**	**Subthemes**	**Description**
Necessity of stakeholder buy-in	a. Ambivalence versus enthusiasm. b. The impact of poor communication.	Stakeholder buy-in and supportiveness improves patient experience, with communication issues around lack of knowledge and role unclarity impacting buy-in. Clear communication and training are needed to engage stakeholders.
A facilitator of meaningful connection	a. Many kinds of connection. b. Connectedness improves wellbeing. c. The ability to connect fosters a sense of agency.	Connection to different spheres (e.g., social vs. educational) held different meanings depending on patient needs. Connectedness facilitated by a robot appeared to reduce patient distress, improve mood, and foster a sense of agency.
One size does not fit all	a. Not always suitable. b. Catering to the unique needs of each patient.	Certain individual and environmental factors may make some patients less likely to benefit from a robot. Service individualization and flexibility is key in meeting individual needs.

#### Theme 1: The Necessity of Stakeholder Buy-in

##### Ambivalence Versus Enthusiasm

The TRECA service is a complex service to implement due to the large number of stakeholders involved (i.e., school principal, teaching staff, school IT team, keyworker, service coordinator, Canteen IT team, patient, and family). It was clear that successful implementation of a robot required buy-in, commitment, and enthusiasm on the part of all stakeholders. This was particularly important when it came to the patient’s school. Patients’ experiences of utilizing their robot tended to be less positive in schools that were unable to dedicate sufficient time to the management of the service (e.g., not having a dedicated teacher to oversee the robot), or with staff that were skeptical or ambivalent about the service and resistant to making accommodations for it in the classroom.

“*if [the school is] not supportive and if they’re not on board, then it’s not going to be effective. That young person’s not going to feel comfortable using it*.” Keyworker

“*We had one teacher tell him to log out and log back in again [at a later point], which I was particularly unhappy about. Because he’s quite happy to sit there and read or do something else until she’s ready for him*.” Parent

This was in stark contrast to schools with staff who were supportive, dedicated resourcing to the robot and were passionate about keeping the patient engaged. These factors resulted in a much more successful and positive experience for the patient.

“*He’s had no issues whatsoever.*… *Because school did so much work prior to [patient] starting to use the robot, I think that had a big impact on it.*… *They’ve just gone, really, above and beyond. He wanted to go to band practice while he wasn’t there physically; they made sure they sent a drum home so that he could still play through the robot. Whatever they can do to make him feel included, they’ve done. I think that’s just really taken any worry off for [patient] and everyone.”* Keyworker

##### The Impact of Poor Communication

School staff and keyworkers were often unclear of what their responsibilities were with regard to contributing to the service, impacting these stakeholders’ abilities to buy into and fully engage with the service.

“*I don’t think anyone was actually really sure who was actually in charge of [the robot]*.” Teacher

Lack of robot software/hardware, usage, or process knowledge also had a significant impact on buy-in, with teachers’ concerns about privacy and unfamiliarity with how to use the robot resulting in resistance to utilizing it in their classroom.

“*I think that one of the downfalls is that teachers don’t necessarily or always know how to use it. Or how to not just use it as in how to make it work – but how to incorporate it in the classroom*” Parent

“*the worry*… *was with privacy issues, the ability to maybe be recorded, or the lessons to be recorded and then who’s going through [those recordings]?*” Teacher

Additionally, communication breakdowns regarding when the robot was going to be used, whose responsibility certain tasks were, and who needed to maintain various aspects of it also led to delays or missed opportunities for the patient to use their robot on a number of occasions.

“*I think there was a problem with engagement, or communication, or maybe someone to be fully assigned to the task of making sure that the robot was in the class at the moment [patient] was going to connect. Because it happened several times, that he was going to connect but the robot wasn’t there – it was not ready for the connection*.” Parent

Finally, a factor impacting buy-in on the part of the patient was the frustration and/or loss of confidence experienced as a result of having to contend with technical difficulties in some of the robots. Although it was acknowledged that technical issues are to be expected with any technology-based service, the impact of not always rectifying these issues in a timely manner on patients’ engagement with the service was highlighted.

“*it was dogged with technical issues and what happened was that, with that, I suppose [patient] lost confidence in using it*… *it was really adding to the stress of everything*.” Parent

As a result of these issues, it was clear that strong and timely communication between stakeholders, and thorough information provision and training of patients, keyworkers, and school staff prior to implementation was needed to fully engage stakeholders in the service.

“*the more training and support that there is for [keyworkers] to just have a general understanding of what’s going on and how to use [the robot], the more empowering that’s going to be for the staff to confidently support the young person*.” Keyworker

#### Theme 2: A Facilitator of Meaningful Connection

Despite the complexity of the set-up and ongoing running of the service, participants consistently reported the value that the TRECA service had in facilitating meaningful connections for patients during a time of their lives where they otherwise feel extremely isolated and disconnected.

##### Many Kinds of Connection

Facilitation of connection to various spheres was reported by participants, with these connections holding different meanings for patients depending on what their values and goals were when entering the service. Participants described the benefit in patients flexibly being able to use their robot more for academic or socialization purposes, depending on individual needs. However, the importance of the social connection to the classroom environment was emphasized over and above that of the academic connection the robot provided.

“*I really loved also just being in class and just listening, not necessarily doing the work, but still just involved and feeling like a part of the group, a part of the class*… *So just keeping me included in my friendship group, my class group, the conversation*” Patient

The breadth of social connection that the robot was able to provide also encompassed connections to the wider school community.

*“It helped me to stay connected and stay close to especially my core group, but not just my core group, all my peers. So just even rolling down the hallway and seeing everyone and just, once again, being in that environment and in that sort of group in collection, it makes you feel like you’re a part of something*” Patient

The robot also provided patients with a sense of connection to their friendship group. One way in which this was facilitated was through assignment of “robot buddies.”

*“[each morning] his friends will come and open the door for [the robot], make sure he can get [it] out [into the hallway], and he’ll go to his lesson with his friends*” Keyworker

Although the facilitation of social connection was the key drawcard of the service for many participants, its ability to enable patients to keep up with their classwork, preventing them from falling behind their cohort, was also seen as a core benefit for many.

“*when I did his initial assessment, the real only stress that was identified in that was around the lack of attendance to school and his drive to do his favorite subjects, so he could get into career path he wanted. So, I think that providing him with that opportunity to attend in an alternative way, was really beneficial for him*” Keyworker

##### Connectedness Improves Wellbeing

A strong link was made by participants between patients’ sense of connection to their peers, school community, and schoolwork, and their wellbeing. The ability for a patient to use their robot appeared to reduce distress or anxiety and improve mood, either because it provided an avenue of social support, or because it prevented the young person from feeling as though they were falling behind on schoolwork.

“*What I have found is that in term three [patient] was connecting and it was really great that he could do that in between treatments, and I think that kept him buoyant in terms of his emotional wellbeing*” Teacher

“*I get really anxious if I’m falling behind. Just to be in the class, just to hear what’s going on, even if I’m not doing work, just eased me a bit*.” Patient

##### The Ability to Connect Fosters a Sense of Agency

Connection to school additionally provided patients with a sense of agency during a time where they have little to no control over their cancer treatment and experience. Using a robot gave patients the choice to take part in the normal day-to-day activities of attending class or talking to their friends, but on their own terms.

“*They’re not in control of the cancer, they’re not in control of the medication that they have to take, so anything that they feel like they can control in their environment is really important and it just gives them that sense of control as well. So they can tune in when they want*” Parent

“*it’s on my terms; it makes me feel like I’m accomplishing something – when I’m having a bad day and I can go into school*.” Patient

#### Theme 3: One Size Does Not Fit All

##### Not Always Suitable

The TRECA service was not suitable for all young patients who met the service’s admission criteria, with certain individual and environmental factors playing a role in reducing potential benefits. For a few young people, concerns about stigmatization and peers’ perceptions of them or the robot impacted significantly on their experience:

“*It was kind of weird especially because I am older and everyone was kind of like, “what is that?,” and then it just made me feel quite uncomfortable to use and I would have rather just been in school. It made me feel like I stood out more than I already did*” Patient

For some patients, rather than helping them to feel more connected, using a robot instead intensified their feelings of disconnection from their school, peers, and friends. Limitations of the robot meant that it was sometimes unable to facilitate as rich of a connection as would be achieved with the patient’s physical presence in some situations. For instance, the robot’s audio capabilities could make hearing what someone was saying difficult when others were talking, and limited mobility meant that full participation at recess or lunchtime was not always possible:

“*I think this is probably one of the biggest issues [that] anyone in this situation at this age would probably say is, “The mates don’t want to hang out in an area that’s going to be quieter.” Because they want to be kicking a ball or something like that*… *And I think he probably feels a little bit left out because of that*.” Teacher

Furthermore, participants reflected on the fact that for some patients, using a robot reminded them of what they were missing out on, and was thus an upsetting experience. As a result, it was suggested that more work could be done prior to implementation in preparing patients for the possibility that they may experience distress when using their robot.

“*I [had been] with that class for about 6 years, and when I walked back in [on my robot] and I wasn’t there in person with them and I couldn’t do stuff with them, it really made me get emotional*… *I was just feeling sad, and I couldn’t do anything. I couldn’t be with my friends*” Patient

Another consideration raised regarding patient suitability for the service was that young people who are disinterested in school prior to their illness may not engage as well with the service in comparison to those who are highly connected to school.

“*I’d say [patient engagement] really depends on the young person and what their attitudes are toward school and why they’re using it. In my experience, most of the young people that have received a robot are pretty passionate about school and, before being diagnosed, were getting really good grades and had a real sense of belonging at their school*… *I can imagine it would be really different for a young person who doesn’t like their school; doesn’t engage well in class*.” Keyworker

A final point regarding suitability that interviewees expressed was that the intensity of treatment and subsequently how unwell the young person became was often a barrier to robot use. For some patients, this inability to use their robot due to illness became a source of distress. Participants spoke about the need to consider whether certain phases of an individual’s cancer journey will be less conducive to robot use than others due to the impact on health.

“*I just feel for me, personally, I was sick in that first couple of phases [to the point] where I couldn’t do anything*. *I felt guilty for just letting it sit there when someone else could have been using it and using it to its actual potential.*” Patient

##### Catering to the Unique Needs of Each Patient

Patients utilizing the TRECA service often had changing needs and preferences, and frequently experienced a highly unpredictable cancer journey. As such, taking an individualized approach when implementing a robot was critical. Participants reported seeing various features of the Kubi^TM^ and Double^TM^ robots as being appropriate for some patients, and not appropriate for others, highlighting the importance of providing patients with the ability to choose the type of robot that will best suit their needs.

The perceived utility of the Double^TM^ robot’s key feature of being able to “drive” around the classroom or school appeared to depend on a number of factors, including the purpose of use, school layout (e.g., presence of stairs), personal preference, and the types of classes it was used for. Generally, participants emphasized the value of this feature for patients who tended to be younger and wanting to use their robot for social connection.

“*I think it [the service] absolutely needs it [the robot] moving around because it makes you feel like you’re actually there, rather than just Skyping in. You can walk around with your friends. It’s just that extra level that just tops it off and makes it amazing. It wouldn’t be the same without moving it, I feel like, and if you were going to work in a different area with [your friends] or sit around or stand up around recess, you get that face-to-face conversation*.” Patient

Others emphasized the benefits of the Kubi^TM^ over the Double^TM^ for patients who are older and only wanting to use a robot solely for academic reasons.

“*I think I’d [prefer to] just have [a robot] that could go on the desk and it could still turn around, but not sort of move around the classroom*… *Once you sit at a desk, you stay there until the period is up, so there’s not really a need, and a lot of people wouldn’t have recess and lunch, I feel like - if they’re older - with their friends. There is not that need to sort of move around.*” Patient

Allowing for further customization of the experience, both the Double^TM^ and Kubi^TM^ provided patients with the option to turn their camera off when connected to their robot. This option can be utilized if patients have concerns about showing their face to their peers and teacher when they are feeling unwell or self-conscious of their physical appearance. Indeed, participants described the value of this function in allowing patients to choose not to be seen, or to simply listen in class rather than join in on discussions and activities, when needed.

*“most of the time I could see everything, but no one could see me because I didn’t want them to, and I didn’t talk, I just listened in*… *[being seen] was just uncomfortable. I didn’t need it, as I said before, I didn’t really need people looking.”* Patient

The utility of having the patient trial the robot prior to full implementation, to further clarify their individual practical needs and preferences for using the robot in the classroom environment, was also reported.

“*the trial was so important, I think, out of everything, and him being able to just go around the school and find out what works for him – if there’s any barriers or anything that won’t work, or we need to adapt or change – while no-one was in the school. That was really great. Even just in his classrooms. The teachers had laid out a spot on the carpet where [patient] could park the robot, and by doing that trial we realized that it was really too far away from the [white]board and we needed to adapt that*.” Keyworker

## Discussion

Given the significant school absences young cancer patients can experience (Tsimicalis et al., 2018), the present study investigated the development and pilot-testing of a telepresence robot service that enables patients to connect to school socially and academically. The Phase I needs assessment and ideas generation demonstrated the need for, and relevance of, telepresence technology in connecting young cancer patients to school and supporting their educational needs. Considering the variable support provided to patients undergoing treatment, a telepresence robot service was perceived as a novel and acceptable method of facilitating a school-patient connection, potentially reducing the impacts of the isolation felt during cancer treatment. The findings and recommendations provided in Phase I, such as the need for ongoing education, training and support to the patient and school, were utilized to inform the development of the TRECA service. Phase II examined the implementation experiences of young people using the TRECA service. Findings from Phase II showed the importance of stakeholder buy-in, the valuable connections that the robots facilitated, and the need for an individualized and adaptable service to meet the varying education and psychosocial needs of adolescents with cancer.

Results from Phase I highlighted the educational and wellbeing impacts that cancer has on adolescents. Participants reported the need for solutions to support patients’ education during cancer treatment, as there was inconsistency in the support available to adolescents during their cancer experience. Participants also highlighted the isolation patients felt during their cancer treatment, as they were not able to connect with their peers in the school environment. Research has shown that prolonged absenteeism from school has impacts not only on cancer patients’ academic attainment but also on their peer relationships (Winterling et al., 2015), leading to social isolation (Tsimicalis et al., 2018) and increased risk of bullying (Collins et al., 2019). A previous study assessing the use of telepresence robot technology with child and adolescent cancer patients found that the technology facilitated both social and educational connection (Weibel et al., 2020). In the present study, the use of telepresence robots was endorsed as an acceptable solution for adolescent cancer patients, with a robot’s dual potential of facilitating education and ameliorating isolation within the school environment.

Recommendations from Phase I highlighted the importance of assessing whether a young person is suitable for the robot’s service. For this reason, the TRECA service includes a referral to a psychosocial support worker (keyworker) who can work with the patient, their family, and the school to develop an implementation plan, defining when and how their robot will be used. The results from Phase I also showed the importance of the implementation of a telepresence robot into the school environment being complemented by ongoing education, training and support for the patient, their family, and the school to ensure the school stays engaged with the robot service. Indeed, previous research suggests that to achieve integration of the telepresence robots in the classroom, school staff training is required to ensure teachers are confident to use the technology (Weibel et al., 2020). General education with a patient’s peers has also been suggested to be protective against bullying of cancer patients by reducing fear of social interaction and improving peer acceptance of the cancer diagnosis (Collins et al., 2019). In addition to education, training, and support, Phase I recommendations also highlighted the necessity of implementing ongoing IT support into a telepresence robot service to facilitate a good user experience with the technology. Research suggests that technology issues with telepresence robots can detract from the positive experience of the telepresence robot, limiting its effectiveness (Weibel et al., 2020). As a result of our findings, we created the TRECA service – an integrated telepresence robot service for adolescent cancer patients. To ensure this service was effective, education and training on the service and cancer was provided to the patient’s teachers and peers, and ongoing IT support was implemented as an essential component.

Results from Phase II highlighted the importance of stakeholder buy-in for the success of a telepresence robot service, and how success can be impeded by insufficient communication. Participants reported a connection between positive patient experiences and school supportiveness, and that greater support and clarity was needed regarding the assignment of responsibility for the robot, how to use the robot, how the service runs, and privacy and technological issue management. The theme of the necessity of stakeholder buy-in further emphasized Phase I’s finding that the implementation of a robot cannot be approached from a standpoint that views the robot itself as the key service component. Rather, the implementation of a telepresence robot should be viewed as a “wraparound” service with many interrelated parts, each of which have a critical role to play in the service’s success. As such, adequately preparing and informing all stakeholders of their role and responsibilities ahead of the implementation day, appears to be key in potentially preventing patient and school staff stress, hesitancy, or disengagement.

These findings on the importance of stakeholder buy-in are supported by previous research by Page et al. (2020) that identified the essential need for planning to occur between stakeholders, and for support and training provision when implementing telepresence robots for chronically ill students. Furthermore, a review by Hinton and Kirk (2015) found that teachers have a lack knowledge of chronically ill students’ healthcare needs, symptom and medication impacts, how they can adapt their teaching to accommodate student needs, and how long-term absence or a students’ return to school should be managed. In addition, both qualitative and quantitative studies have found that teachers lack confidence in meeting the needs of students with illness and are concerned about risks and responsibilities associated with supporting these students (Wilkie, 2012; Hinton and Kirk, 2015). As such, patient and teacher experience may be enhanced by providing teachers with information about the purpose of the robot in the classroom, how they can effectively incorporate the robot into their teaching, and what their student’s needs and preferences are. Guidelines and resources advising teachers on how the robot’s hardware and software works, along with consistent provision of accessible and timely support, may also give patients and schools greater confidence in knowing how to manage the inevitable technical difficulties that arise in any technology-based service. Similarly to the present study, Page et al. (2020) also reported teachers’ concerns about privacy impacted their willingness to use a robot. As such, ensuring adequate and accessible information is provided to schools about how the service mitigates common teacher concerns such as privacy and impact on workload may also improve school buy-in, and in turn, the patient’s experience.

Another key finding from Phase II was the notion that the TRECA service was a facilitator of many kinds of meaningful connection, and that this connectedness underlay wellbeing and fostered a sense of agency for the young person. It was evident that some patients tended to choose to use their robot more for social connection purposes rather than academic reasons, and vice versa. The individualized nature of the TRECA service allowed facilitation of the kind/s of connection each individual patient valued based on their needs, preferences, and cancer experience. Indeed, given the TRECA service’s core purpose is to improve patient wellbeing, this finding suggests that facilitating the kind of connection that is of most value to the individual patient, in the way that is most comfortable for them, is likely to result in the greatest benefit. Patient preference for a certain type of connection over another was also found by Weibel et al. (2020), who reported that incongruence between student needs and teachers’ understandings of the purpose of the robot and how the patient wished to participate at school (preference for academic rather than social use) created a barrier. Phase II findings also showed that participants’ preference for and use of various robot features (e.g., mobility, allowing face to be shown on-screen) differed from individual to individual based on their needs, further illustrating the importance of a flexible telepresence robot service. Interestingly, although previous research has suggested that mobile telepresence robots are likely more appropriate and better facilitate connectedness in a school context than stationary robots (Page et al., 2020), the present findings suggest that robots like the Kubi^TM^ (which is stationary but allows the user to swivel their viewpoint) may be a better fit for the needs of older students who are tending to use a robot more for academic reasons.

Results suggested that the social and academic connections facilitated by the robot appeared to improve patients’ moods and/or reduce feelings of stress due to the way it enabled them to socialize or participate in schoolwork. These findings align with previous research. For instance, perceived social support has been negatively related to depression and anxiety in adolescents with leukemia (Çavuşoğlu and Sağlam, 2015), and school connectedness has been found to be predictive of future psychosocial adjustment in child and adolescent cancer survivors (Okado et al., 2018). The TRECA service also appeared to provide patients with a sense of agency and achievement during a time in their life that was otherwise void of choice. These results align with research demonstrating telepresence robots’ positive impacts on the wellbeing and sense of autonomy of school students with a chronic illness (Page et al., 2020). Importantly, Belizzi et al. (2012) found many adolescent and young adult cancer patients report that “control over life” is a core aspect of life that is negatively impacted by cancer. Given loss of control has been linked to poor psychological and treatment adherence outcomes in child and adolescent cancer patients (Wicks and Mitchell, 2010), it has accordingly been advised that clinicians try to foster patients’ sense of control in areas of their lives they are able to have control over (e.g., health promoting activities; Belizzi et al., 2012). As such, a telepresence robot service may be of great benefit in enabling patients to feel they have control and choice over their schooling and socialization.

Finally, the results from Phase II highlighted that a telepresence robot is not suitable for all adolescent patients being treated for cancer. Prior to a patient’s acceptance into a telepresence robot service, it is recommended that the client’s needs are adequately understood, and it is ensured their needs can be met by the service. For instance, is the kind of robot they prefer available? Are their expectations of the service realistic? Are they motivated to “attend school” and well enough to use a robot? Furthermore, providing psychoeducation on the benefits and potential negatives associated with using a robot (e.g., experiencing feelings of disconnectedness rather than connectedness) may enable protective factors to be put in place ahead of implementation. Service-users would also likely benefit from school and peer supportiveness being assessed prior to implementation. Where significant needs, barriers or concerns are identified through this screening process, psychosocial staff may be able to work with patients and schools to address these issues and ensure that schools are aware of patients’ preferences. As evidenced in Phase II, a pre-implementation robot trial also provided valuable feedback on patient needs and preferences and identified practical barriers. A trial period may additionally assist with setting patient expectations and normalizing encountering of unavoidable hurdles such as the patient being too sick to use the robot some days or technological difficulties.

### Recommendations for Implementing a Telepresence Robot Service

The pilot study allowed participants to provide insights into their experience of using a telepresence robot (Table 3). These valuable insights will be used to further refine and strengthen the TRECA service. The provision of ongoing information, training and support can facilitate stakeholder buy-in, ensuring sustained engagement with the service from patients, parents, and teachers. The identification and management of potential barriers to suitability and success with potential patients prior to the implementation of the robots’ service is also recommended.

**TABLE 3:** Recommendations for implementing a telepresence robot service.

**Domain**	**Recommendation**
Initial information provision and training	– Chart outlining all stakeholder responsibilities and when/how they should be contacted.
	– Website housing all TRECA documents andresources.
	– Videos explaining the service in detail, demonstratinghow to use and troubleshoot the robot.
	– Psychosocial support worker training anddocumentation on their role, how the service works, and the responsibilities of all stakeholders.
Specialized teacher training/information	– Video resources showing other teachers’ experiences of the robot and tips for incorporating it into teaching method.
	– Patient preference survey given prior toimplementation, indicating how patients would like toparticipate using their robot.
Technical information provision, training, and support	– Video resources showing other teachers’ and schools’experiences with technical difficulties or privacyconcerns that arose; video and written resources thatcan be referred to as needed showing how the robotworks, and how to troubleshoot basic problems theymay encounter.
	– Increasing staffing as the service grows to ensureresponse is as fast as possible. If a tech issue persists, consider replacing the patient’s robot andtroubleshooting the problem in-house so the service isimpacted as little as possible.
Identification and management of potential barriers to suitability and success	– Formalizing the intake process to ensure key factorsindicating suitability are assessed.
	– Providing psychoeducation to patients about potentialfor distress to be experienced, and putting in place aplan to manage concerns/potential for distress withkeyworker as needed.
	– Providing psychoeducation to patients normalizingencountering of common barriers such as being too illto use the robot sometimes.
	– Completing assessment of school and peersupportiveness with school prior to implementation;putting in place a plan to address non-supportivenessas needed.
	– Offering a pre-implementation trial to every patient.
Ongoing communication with the school	– Implementing a standardized system forcommunication between school and patient regardingattendance (e.g., app, online interactive timetable).

### Strengths and Limitations

The strengths of the present study were its use of a PD process, which ensured that the telepresence robot service’s planning, design, and implementation were guided by young cancer patients, parents, teachers, healthcare workers and psychosocial clinicians. In both phases of the study, participation from key stakeholders also ensured that a full picture of the impacts of and issues surrounding the service could be established. Limitations of this study included the potential that response bias may have occurred, where participants recruited to Phase I and II were those who tended to have a better experience with the service or more positive view of a robot service, and as such had a greater desire to take part in the study. Additionally, only young cancer patients and their parents, teachers, keyworkers and healthcare workers were included in Phase II’s pilot testing, and as such, conclusions cannot be drawn about how a robot service may suit and benefit other populations (e.g., those with other illnesses or reasons for missing school).

### Policy Implications and Future Directions

Using a PD framework (Clemensen et al., 2007), this two-phase study used interviews to assess stakeholders’ views on using a telepresence robot service to connect cancer patients to school and understand stakeholder experiences of a subsequent pilot-test of this service. Phase I identified key stakeholder needs, potential service barriers and facilitators, and found high perceived acceptability. In Phase II, key themes were generated from interviews regarding the importance of stakeholder buy-in, facilitation of meaningful connection, and a telepresence robot service not being one-size-fits-all. The TRECA service’s ability to be individualized and flexible in meeting patient needs enabled young cancer patients to connect academically and/or socially to their schools. Participants reported that engagement with the service appeared to enhance patients’ sense of agency and wellbeing. The positive impact of a telepresence robot service has highlighted the need to consider how such a model may be embedded more broadly across the education system in Australia. In particular, we recommend that information about the service become commonplace amongst educators in Australia, with tools and information available to them to both offer this option as a point of referral for their students in the case of a cancer diagnosis; and, to decrease barriers of participation amongst the educators themselves. By applying stakeholders’ recommendations from the present study to improve existing processes and procedures, the TRECA service can continue to grow in effectiveness and capacity.

## Data Availability Statement

The data that support the findings of this study are not publicly available as they contain potentially identifying and sensitive information. No ethical approval was obtained for the sharing of this dataset with researchers external to this project. Contact the corresponding author for further information.

## Ethics Statement

The study was reviewed and approved by the University of Sydney and University of New South Wales Ethics Committees. Written informed consent to participate in this study was provided by the participant or a legal guardian/next of kin.

## Author Contributions

PP and JC contributed to the study design. TP collected the participant data. TP and JC analyzed the participant data. TP, JC, and PP prepared the manuscript. All authors read and approved the final manuscript.

## Conflict of Interest

All authors are affiliated with Canteen. Canteen provides and owns the intellectual property rights to the TRECA service described in this paper, including any potential financial benefits that may result from future service provision.

## Publisher’s Note

All claims expressed in this article are solely those of the authors and do not necessarily represent those of their affiliated organizations, or those of the publisher, the editors and the reviewers. Any product that may be evaluated in this article, or claim that may be made by its manufacturer, is not guaranteed or endorsed by the publisher.

## Footnotes

1 Canteen is the national Australian organization supporting young people (12–25 years) impacted by their own or a family member’s cancer diagnosis.

## Abbreviations

ICT: information and communication technology
TRECA: telepresence robots to engage cancer patients in education
PD: participatory design.
