# Supplementary material for: Keeping Connected With School: Implementing Telepresence Robots to Improve the Wellbeing of Adolescent Cancer Patients
Source: Front Psychol. 2021 Nov 12;12:749957. doi: 10.3389/fpsyg.2021.749957 (PMC8636051; doi:10.3389/fpsyg.2021.749957)
Supplement: Supplementary file 1 [file Data_Sheet_1.docx]

Appendix 1: Phase I Interview Schedule

| **Group** | **Interview Questions** |
| --- | --- |
| **Patient/Family** | **Needs Assessment**   1. Tell me about your experience of receiving hospital treatment as part of your/your child’s cancer treatment plan 2. How much school did you/your child miss due to treatment? 3. How is/did your child’s absence from home and/or school impact on the family? 4. To what extent did you/your child keep in contact with the school while you were absent? 5. Were you/your child offered any academic support 6. What other kind of support did you want/think would be helpful? 7. Did you/your child keep in touch with your friends while you were off school 8. What was your/your child’s experience of returning to school OR what are your/your child’s thoughts about returning to school   **Ideas Generation**   1. How easy/difficult do you think it to use the robot? 2. What did you think of the camera? Would you be happy to use it? 3. What are your thoughts on the visual look of the robot? 4. Would you/your child you have liked to use this robot during the cancer experience? 5. Are there any additional things you would require to be able to use this robot in school/at home? |
| **Teacher** | **Needs Assessment**   1. Have you ever had a student who missed a lot of school due to a (chronic) illness? How did you support them? 2. Did the student remain engaged with schoolwork? Friends at school? School community? 3. When the young person returned to school, how did peers in respond? 4. Whose responsibility do you believe it is to keep adolescents engaged with school/schoolwork if they are unwell? 5. What is the best way to engage absent students?   **Ideas generation**   1. How do you think a robot would work in your school? 2. What would stop this technology from working in your school? 3. What would help technology to work in your school? 4. Do you feel the robot was easy to use? |
| **Healthcare Worker** | **Needs Assessment**   1. What impact do you think being hospitalized has on a young person? 2. What do you think are the social, emotional, and educational needs of a young person whilst they are in hospital? 3. Are there any rules surrounding the use of technology by patients/family in the hospital? 4. Is any educational support provided to patients aged 12-18 years?   **Ideas Generation**   1. How do you think a robot like this would work with your patients? 2. What would stop this technology from working in your hospital? 3. What would help technology to work in your hospital? 4. Are there aspects of the Robot that you think would be particularly beneficial for young people? |
