# Supplementary material for: Keeping Connected With School: Implementing Telepresence Robots to Improve the Wellbeing of Adolescent Cancer Patients
Source: Front Psychol. 2021 Nov 12;12:749957. doi: 10.3389/fpsyg.2021.749957 (PMC8636051; doi:10.3389/fpsyg.2021.749957)
Supplement: Supplementary file 2 [file Data_Sheet_2.docx]

Appendix 2: Phase II Interview Schedule

| **Group** | **Interview Questions** |
| --- | --- |
| **Patient** | 1. How often have you been using the robot to connect into school? 2. What classes have you used it in? 3. Do you use the robot to talk to your friends? 4. Do you think you’ve completed more school/homework since using the robot than you would have if you didn’t have it? 5. As time goes on, have you been using the robot more or less?? 6. (If finishing the program and returning the robot to Canteen) Why have you decided to stop using the robot? 7. Do you think using the robot has been a good experience for you? 8. What are the best things about using the robot? 9. What are the worst things about using the robot? 10. Has the robot had any influence on your friendships? 11. Has using the robot changed how you feel about school? 12. How easy has it been to connect into your classes through the robot? 13. What is working well about the robot? 14. What isn’t working about the robot? 15. Is there anything you have discovered that makes using the robot easier/better? 16. Is there anything you think Canteen could do to make using the robot better? 17. Is Canteen providing you with enough support and technical help? 18. Would you recommend the robots program to other young cancer patients? |
| **Parent** | 1. How is your child’s treatment impacting their ability to engage with school and their robot? 2. In your opinion, how do you think your child has been going with the robot? 3. Are they using it as often as you expected them to? 4. How does your child use their robot? 5. How motivated and engaged do you believe your child is with using their robot? 6. Has using a robot been a good, bad, or mixed experience for your child 7. What are the best things about your child using the robot? 8. What are the worst things about your child using the robot? 9. Do you think your child has benefited from using the robot? 10. What sort of impact, if any, has the robot had on your child’s friendships? 11. In terms of the technology, how easy has it been for your child to use the robot? 12. What is working well about the robot? 13. What isn’t working about the robot? 14. Is there anything you and [patient] discovered that makes using the robot easier/better? 15. Is there anything the school has done to help with robot that has been beneficial? 16. Is there anything you think the school could do further to make using the robot a better or easier experience? 17. Is there anything you think Canteen could do to make using the robot a better or easier experience? 18. Is Canteen providing you and your child with enough support and technical help? 19. Would you recommend the robots program to other families with a child with cancer? |
| **Teacher** | 1. What sort of impact has [patient]’s cancer had on their school experience? 2. Before the robot was implemented at your school, how was the school supporting [patient] after their diagnosis? 3. In your opinion, how do you think [patient] has been going with the robot? 4. Are they using it as often as you expected them to? 5. How does [patient] use their robot at school? 6. How motivated and engaged do you believe [patient] is with using their robot? 7. In your opinion, has having a robot been a good, bad, or mixed experience for the school, yourself and other teachers? 8. What are the best things about the robot? 9. What are the worst things about the robot? 10. Do you think [patient] has benefited from using the robot? 11. Do you think that using the robot has changed the way [patient] relates to school? 12. In terms of the technology, how easy has it been for you and other staff to manage the robot at school? 13. Does anything ever get in the way of the patient being able to attend or engage with the class? 14. How often do you experience issues with the robot? 15. What is working well about the robot? 16. What isn’t working about the robot? 17. Is there anything you or other staff have discovered that makes having the robot in the classroom easier or better? 18. Is there anything you think Canteen could do to make having the robot in your classroom/school better or easier? 19. Is Canteen providing your school with enough support and technical help? 20. Would you recommend the robots program to other schools supporting a student with cancer? |
| **Keyworker** | 1. In your opinion, how do you think [patient] has been going with the robot? 2. Are they using it as often as you expected them to? 3. How motivated and engaged do you believe [patient] is with using their robot? 4. Are you aware of anything ever getting in the way of the patient being able to use their robot? 5. In your opinion, do you think having a robot has been a good, bad or mixed experience for the [patient]? 6. What do you think the best things about the robot are for [patient]? 7. What do you think the worst things about the robot are for [patient]? 8. Do you think [patient] has benefited from using the robot? 9. Do you think that using the robot has changed the way [patient] relates to school? 10. Do you think that using the robot has changed [patient]’s social connectedness? 11. In terms of the technology, how easy has it been for [patient] to use the robot? 12. Does [patient] ever contact you with technological issues they are experiencing with the robot? How often? What issues do they seem to have? 13. What is working well about the robot for [patient]? 14. What isn’t working about the robot for [patient]? 15. What are your thoughts on how the robot was initially implemented by Canteen? 16. Are there any ways in which you believe the implementation process can be improved? 17. Have there been any communication issues within and external to Canteen in supporting [patient] with their robot? 18. How clearly defined do you believe your role in supporting [patient] with their robot is? 19. Are there any ways in which you believe Canteen’s support of [patient] can be improved? 20. Did Canteen provide you with enough support and technical help? |
